# Thyroid Eye Disease due to Immune Reconstitution Inflammatory Syndrome as a Consequence of Antiretroviral Therapy in the Setting of AIDS

**DOI:** 10.1155/2020/1728423

**Published:** 2020-02-12

**Authors:** Ravali Nallu, Parvathy Madhavan, Lisa Chirch, Pooja Luthra

**Affiliations:** ^1^Department of Medicine, Division of Endocrinology and Metabolism, University of Connecticut, School of Medicine, Farmington, CT, USA; ^2^Department of Medicine, Division of Infectious Diseases, University of Connecticut, School of Medicine, Farmington, CT, USA

## Abstract

We describe a case of worsening Graves' orbitopathy due to immune reconstitution inflammatory syndrome (IRIS) in a 38-year-old HIV-infected male after beginning ART (antiretroviral therapy). Two years after initiation of ART, the patient developed symptoms of hyperthyroidism and thyroid eye disease (TED) or Graves' orbitopathy (GO). Thyroid iodine uptake scan was consistent with Graves' disease. The CT scan of the orbits revealed minimal right-sided proptosis, consistent with GO. He was treated with methimazole and a short course of high-dose prednisone for GO. Thyroid function tests normalized, and eye symptoms eventually stabilized. This case demonstrates the importance of awareness and early recognition of IRIS in its many forms, as it has significant therapeutic implications.

## 1. Background

Antiretroviral therapy (ART) has dramatically improved the morbidity and mortality of patients infected with the human immunodeficiency virus (HIV), but it may also increase the risk of immune reconstitution inflammatory syndrome (IRIS). IRIS is characterized by a pathological inflammatory response to a pre-existing pathogen or antigen and paradoxical worsening of clinical status, after initiation of ART [1]. IRIS develops in 10–25% of patients with AIDS receiving ART [1]. Graves' disease (GD) occurs in 1-2% of patients with HIV as a late manifestation of IRIS [2]. GD is an autoimmune disease caused by production of IgG autoantibodies against the thyrotropin (TSH) receptor, binding and activating it and causing autonomous production of thyroid hormone [3]. Thyroid eye disease (TED) or Graves' orbitopathy (GO) occurs in about 25% of all patients with GD [1]. The incidence and prevalence of TED as a manifestation of IRIS is unknown. We discuss the rare occurrence of TED due to GD, as a result of IRIS, in a patient with AIDS.

## 2. Case

A 38-year-old male diagnosed with HIV-1 infection in 2014 was treated with ART. He did not have a prior history of thyroid disease although his sister had a history of hyperthyroidism. Physical examination was unremarkable. His baseline CD4+ T-cell count was very low at 32 cells/*µ*l with a plasma HIV RNA of 213,000 copies/ml. The patient was started on ART with emtricitabine/rilpivirine/tenofovir disoproxil fumarate at an outside facility. On evaluation at our institution for a second opinion, he was transitioned to emtricitabine/tenofovir disoproxil fumarate, darunavir, ritonavir, and dolutegravir based on resistance testing that revealed reverse transcriptase mutations at the M184 and Y181 sites. This regimen resulted in complete suppression of HIV RNA within the first 12 weeks of therapy, accompanied by a significant rise of CD4+ T-cell count.

At presentation, the TSH level was normal at 1.34 mIU/l (0.27–4.20 mIU/l). Thyroid antibody levels were not available at that time.

Two years after initiation of ART (2016), the patient developed insomnia, palpitations, heat intolerance, unintentional 20 lbs weight loss, and bulging of the eyes. On exam, significant bilateral exophthalmos was noted, along with a prominent thyroid gland.

See Table 1 for laboratory data.

The thyroid iodine I-131 uptake scan showed diffuse increase in the uptake without evidence of hot or cold nodules.

The patient was diagnosed with GD with significant TED and started on antithyroid therapy with methimazole and a beta-blocker. The patient stopped the antithyroid medication after a few months and noticed further progression of eye symptoms. A CT scan of the orbits revealed minimal right-sided proptosis consistent with TED (Supplementary Materials [Supplementary-material supplementary-material-1]). He was restarted on the methimazole and a short course of high-dose prednisone for TED. Thyroid function tests normalized, and eye symptoms eventually stabilized.

## 3. Discussion

HIV affects the immune system by specifically infecting the CD4+ T-cells, resulting in the development of a wide variety of opportunistic infections. Since the development and widespread use of highly active ART in the 1990s, there has been a significant reduction in the morbidity and mortality associated with the disease. However, a small subset of patients treated with ART, in particular those with very low nadir CD4+ T-cell counts, experience a paradoxical clinical deterioration associated with the recovery of the immune system. This has been described as an entity known as IRIS and is believed to affect about 10–40% of patients beginning ART [4]. This presentation is believed to be related to the recovery of immune function and restoration of the ability to mount an inflammatory response to both infectious and noninfectious agents. The exact mechanism is not completely understood. Resurgence of autoimmune conditions has most commonly been shown to occur in the later stages of AIDS [5].

GD as a consequence of IRIS after initiation of ART is well described [6]. It is thought to behave similarly to “conventional” GD and therefore should be treated in a similar manner. However, extrathyroidal manifestations of GD, such as TED, are not well characterized. TED is an inflammatory condition affecting the orbital tissue and occurs in about 25% of patients with GD [1]. It is characterized by lymphocytic infiltration of the extraocular muscles and orbital adipose tissue, orbital fibroblast proliferation and differentiation, deposition of hyaluronic acid and glycosaminoglycans, and de novo adipogenesis [7]. The pathogenesis of this condition is believed to be secondary to a complex interplay between genetic and lifestyle factors (e.g., cigarette smoking) leading to an inflammatory cascade, with involvement of autoantibodies such as TSH receptor (TSH-R) and insulin-like growth factor-1 (IGF-1) receptor [8].

TED has a spectrum of clinical manifestations and can range from mild disease to severe extraocular muscle dysfunction and vision loss due to exposure keratopathy or optic nerve compression [8]. ART treatment increases the CD4+ T-cell count in 2 phases: initial increase in the memory CD4+ T-cells that occurs in the first few months of therapy; and a slower and steady increase over many months to years, in the naive CD4+ T-cells, which originate from the thymus [9]. The CD4+ T-cells mature in the thymus, and cells which are capable of recognizing the major histocompatibility complex (MHC) molecules associated with foreign antigens enter the periphery, whereas those reacting with self-antigens undergo apoptosis [10]. However, initiation of ART causes intense regeneration of the thymus, resulting in an insufficient suppression of the autoreactive cells. This leads to the manifestation of a wide variety of autoimmune phenomenon including GD. Even though the functionality of the thymus is thought to decrease with age, recent reports have shown that it may remain active longer in HIV-infected individuals [11].

Another hypothesis for expansion of autoreactive T lymphocytes and their redistribution in IRIS is due to “molecular mimicry” due to homology between TSH-R and immunogenic HIV-related proteins [8]. Other predisposing factors associated with the development of IRIS include advanced immunodeficiency at baseline, more pronounced increase in CD4+ T lymphocytes, and genetic susceptibility [5].

In 2000, Jubault et al. [12] published a series of five patients, median age of 41 years, who developed GD after the initiation of ART. None of the patients had prior autoimmune conditions. In 2009, Vos et al. [13] described 3 similar cases and performed a literature review that revealed 13 reported cases of GD secondary to IRIS. Wong et al. [14] demonstrated a similar course in 13 HIV-infected patients of Chinese origin. In all of these studies, there was a direct relationship between the rise of the CD4+ T-cell count and corresponding suppression of the viral load, after beginning therapy with ART, and occurrence of GD. This was clearly demonstrated in our case as well. Chen et al. [15] published a cohort of 17 HIV-affected patients, predominantly African American, who developed autoimmune thyroid disease (AITD) as a late complication of IRIS, after treatment with ART.

Management of TED can be challenging in the presence of HIV infection. Patients with poorly controlled thyroid function have more severe manifestations of TED [16]. In addition, certain medications increase the possibility of thyroid dysfunction. In a large single-center cohort of HIV-infected patients, the prevalence of clinically apparent hyperthyroidism was 1.01%, most commonly associated with use of non-NRTI (NNRTI), particularly efavirenz [17]. On the other hand, there was a high prevalence of subclinical hypothyroidism, with the use of nucleoside reverse transcriptase inhibitor (NRTI), stavudine [18]. It is uncertain if these medications increase the severity of TED, but these associations should be kept in mind when choosing ART regimens. It is also important to consider interactions between ART and medications used in patients with TED. NNRTIs may induce cytochrome P450 (e.g., efavirenz), whereas ritonavir-boosted protease inhibitors (PI) inhibit cytochrome P450. Ritonavir and cobicistat (a pharmacologic booster) can significantly increase the concentration and effect of glucocorticoids, potentially leading to Cushing's syndrome [19]. It is important to closely monitor patients with HIV infection on high-risk medications for any manifestations of IRIS for prompt diagnosis and management.

## 4. Conclusion

Our case demonstrates the importance of being vigilant about the occurrence of various autoimmune conditions as manifestations of IRIS in patients receiving ART. These may occur even 1–3 years after initiation of therapy, as described in our case [10]. The management of GD and TED in the presence of HIV infection and ART appears to be equivalent to standard treatment with particular attention to potential drug interactions. Timely recognition and treatment may help to reduce the complications associated with these disorders.

## Figures and Tables

**Table 1:** Laboratory data.

	2014 (Pre-ART)	2016 (Post-ART)	2017	2018
TSH	1.34	<0.01	1.06	0.54
Free T4	N/A	2.38	0.99	N/A
TT3	N/A	526	139	128
TSI	N/A	>500%	N/A	N/A
CD4	32	396	343	344

TSH: 0.35–4.94 mIU/ml. Free T4:0.61–1.82 ng/dl. Total T3 (TT3): 48–178 ng/dl. Thyroid-stimulating immunoglobulins (TSI) < 122%. CD4+ T-cells, 430–1800 cells/*µ*l; N/A, not available.

## Supplementary Materials

Supplementary Materials Image of the CT scan of the orbit.
