# Supplementary figures and images for: Thyroid Eye Disease due to Immune Reconstitution Inflammatory Syndrome as a Consequence of Antiretroviral Therapy in the Setting of AIDS
Source: Case Rep Endocrinol. 2020 Feb 12;2020:1728423. doi: 10.1155/2020/1728423 (PMC7037486; doi:10.1155/2020/1728423)

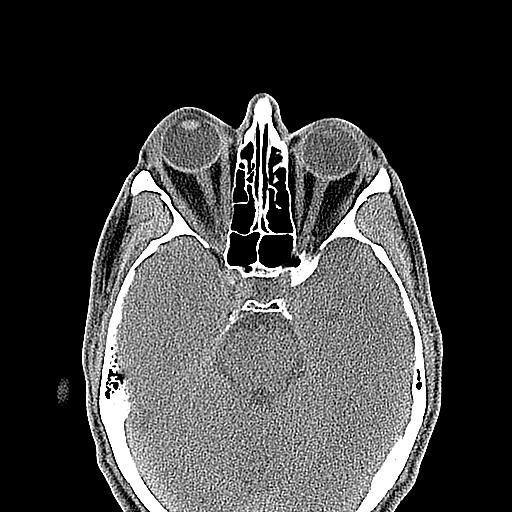

Supplement: Supplementary Materials — Image of the CT scan of the orbit. [file 1728423.f1.jpg]
